# Last Word on Viewpoint: Do we need to change the guideline values for determining low bone mineral density in athletes?

**DOI:** 10.1152/japplphysiol.00227.2022

**Published:** 2022-05-01

**Authors:** Kristin L. Jonvik, Monica K. Torstveit, Jorunn K. Sundgot-Borgen, Therese Fostervold Mathisen

**Affiliations:** ^1^Department of Physical Performance, Norwegian School of Sport Sciences, Oslo, Norway; ^2^Department of Sport Science and Physical Education, University of Agder, Kristiansand, Norway; ^3^Department of Sports Medicine, Norwegian School of Sport Sciences, Oslo, Norway; ^4^Faculty of Health, Welfare and Organisation, Østfold University College, Oslo, Norway

**Keywords:** bone mineral density, bone stress injury, fractures, low energy availability, Z-scores

We appreciate the engagement in the Commentaries (1) for our recent Viewpoint paper (2).

Although our concern related to the use of population normative Z-scores mainly referred to potentially overlooking symptoms of low energy availability (LEA) in high-impact (HI) sport athletes, also overlooking athletes at high risk for fractures is worrying. About 10% of all sports-related injuries, being as high as 30% in running, are stress fractures, depriving athletes from sport participation (3). Tolerance for sport-specific training may take as long as 6 mo postfracture, and more likely up to 12 mo (4). The inappropriate use of bone mineral density (BMD) Z-scores from the general population to evaluate risk in athletes was highlighted by the recent findings of “normal clinical” BMD values in collegiate distance runners who experienced bone stress injuries (5). Heikura et al. (6) reported a 4.5 times higher risk for bone stress injuries in runners and race-walkers with symptoms of LEA, despite “normal” mean BMD Z-scores, which also were similar to the BMD Z-scores of the athletes without fractures (6). Importantly, normal BMD Z-scores, when being based on the general population, do not seem to successfully identify athletes at high risk for bone stress injuries. The difficulties in identifying athletes at risk for complications from LEA, like bone stress injuries, by comparing BMD values to the general population, were also illustrated by Ackerman (7). BMD Z-scores in the femur and the total hip regions were both higher in eumenorrheic runners compared with nonathletes, but also much lower in amenorrheic compared with both eumenorrheic runners and nonathletes (7). This study was the first to report deteriorated microarchitecture in *adolescent* amenorrheic athletes; an important determinant for fracture risk.

Interestingly, DXA does not distinguish cortical from trabecular bone mass, whereas trabecular bone mass is early to deteriorate with persistent overload or poor recovery, the BMD changes may be masked in cortical-dominated bone areas (8). This was highlighted in a finding of similar tibial-BMD in sport- and age-matched females with or without fracture, but with dissimilar regional bone quality (8). Not all studies including bone quality measurements have identified differences between individuals with or without fracture (9). Still, region-specific measurements and consideration of the timing of measurement are important factors that may explain the different findings related to BMD and microarchitecture.

As such, “normal” BMD Z-scores, still being lower than expected for a HI athlete, may indicate a high risk for bone stress injury, and some of the explanations may be impaired bone quality. This is not measurable by DXA but has typically been reported by BMD Z-scores around “0” or less. As long as values above −1 are considered clinically normal, we may overlook athletes at high risk for fractures, also leaving them at high risk for the many other clinical complications by relative energy deficiency in sport (RED-S), if the reason for impaired bone quality relates to LEA. Hence, in contrast to van Weijer et al. (1), we argue that an athlete-specific BMD range may both reduce complications from undiscovered LEA as well as risk for fractures.

## DISCLOSURES

No conflicts of interest, financial or otherwise, are declared by the authors.

## AUTHOR CONTRIBUTIONS

T.F.M. drafted manuscript; K.L.J., M.K.T., J.K.S.-B., and T.F.M. edited and revised manuscript; K.L.J., M.K.T., J.K.S.-B., and T.F.M. approved final version of manuscript.

## References

[1] Weijer V, Hilkens L, Brinkmans N, van Dijk JW, Srivastav S, Gadhvi MA, Fernandes RJ, Filho DP, Santos MP. Commentaries on Viewpoint: Do we need to change the guideline values for determining low bone mineral density in athletes? J Appl Physiol (1985). doi:10.1152/japplphysiol.00203.2022. PMC9208432 35587118

[2] Jonvik KL, Torstveit MK, Sundgot-Borgen JK, Mathisen TF. Do we need to change the guideline values for determining low bone mineral density in athletes? J Appl Physiol (1985). doi:10.1152/japplphysiol.00851.2021. 35060767 PMC9126212

[3] Song SH, Koo JH. Bone stress injuries in runners: a review for raising interest in stress fractures in Korea. J Korean Med Sci 35: e38, 2020. doi:10.3346/jkms.2020.35.e38. 32103643 PMC7049623

[4] Popp KL, Ackerman KE, Rudolph SE, Johannesdottir F, Hughes JM, Tenforde AS, Bredella MA, Xu C, Unnikrishnan G, Reifman J, Bouxsein ML. Changes in volumetric bone mineral density over 12 months after a tibial bone stress injury diagnosis: implications for return to sports and military duty. Am J Sports Med 49: 226–235, 2021. doi:10.1177/0363546520971782. 33259223

[5] Carbuhn AF, Yu D, Magee LM, McCulloch PC, Lambert BS. Anthropometric factors associated with bone stress injuries in collegiate distance runners: new risk metrics and screening tools? Orthop J Sports Med 10: 23259671211070308, 2022. doi:10.1177/23259671211070308. 35178462 PMC8844446

[6] Heikura IA, Uusitalo ALT, Stellingwerff T, Bergland D, Mero AA, Burke LM. Low energy availability is difficult to assess but outcomes have large impact on bone injury rates in elite distance athletes. Int J Sport Nutr Exerc Metab 28: 403–411, 2018. doi:10.1123/ijsnem.2017-0313. 29252050

[7] Ackerman KE, Nazem T, Chapko D, Russell M, Mendes N, Taylor AP, Bouxsein ML, Misra M. Bone microarchitecture is impaired in adolescent amenorrheic athletes compared with eumenorrheic athletes and nonathletic controls. J Clin Endocrinol Metab 96: 3123–3133, 2011. doi:10.1210/jc.2011-1614. 21816790 PMC3200253

[8] Schnackenburg KE, Macdonald HM, Ferber REED, Wiley JP, Boyd SK. Bone quality and muscle strength in female athletes with lower limb stress fractures. Med Sci Sports Exerc 43: 2110–2119, 2011. doi:10.1249/MSS.0b013e31821f8634. 21552163

[9] Rudolph SE, Caksa S, Gehman S, Garrahan M, Hughes JM, Tenforde AS, Ackerman KE, Bouxsein ML, Popp KL. Physical activity, menstrual history, and bone microarchitecture in female athletes with multiple bone stress injuries. Med Sci Sports Exerc 53: 2182–2189, 2021. doi:10.1249/MSS.0000000000002676. 33831898 PMC8440446

